# Assessment of agricultural biomass residues to replace fossil fuel and hydroelectric power energy: A spatial approach

**DOI:** 10.1002/ese3.462

**Published:** 2019-09-09

**Authors:** Cristhy Willy da Silva Romero, Mauro Donizeti Berni, Gleyce Kelly Dantas Araujo Figueiredo, Telma Teixeira Franco, Rubens Augusto Camargo Lamparelli

**Affiliations:** ^1^ School of Agricultural Engineering FEAGRI University of Campinas, UNICAMP Sao Paulo Brazil; ^2^ Interdisciplinary Center on Energy Planning NIPE University of Campinas UNICAMP Sao Paulo Brazil; ^3^ Department of Process Engineering (DEPRO) School of Chemical Engineering University of Campinas, UNICAMP Sao Paulo Brazil

**Keywords:** biogas, eucalyptus, spatial analyses, sugarcane

## Abstract

Despite the recent discoveries of considerable fossil fuel reserves, Brazil is one of the only great economic and industrial powers with very high amounts of renewable energy in its electricity matrix. Approximately 79.3% of the electric energy supply comes from renewable resources, of which hydroelectric power represents 70.6%. The two primary concerns regarding hydroelectricity are the damage caused to the environment by the construction of dams and the uncertainty of the supply in cases of long drought seasons. This article presents an analysis on the availability and energy exploitation of sugarcane straw and forest residues derived from eucalyptus for decentralized generation using a Geographic Information System–based model. The potential bioelectricity and bioethanol production from sugarcane and eucalyptus biomass in the Administrative Region of Campinas (ARC) is higher than the demand in this region. The results provide guidelines for designing alternatives to the intended Nationally Determined Contributions in Brazil within the scope of the ARC, and they can be used to provide energy empowerment, electric matrix diversification, and new policies that address the residue availability and demand.

## Graphical abstract

Assessment of agriculture biomass residues to replace fossil fuels and hydroelectric power energy – A spatial approach.

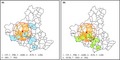

## 1 INTRODUCTION

Because of the impacts caused by extreme events associated with increased levels of carbon dioxide (CO₂) and other gases,^1^, ^2^ people around the world have been discussing how to address this issue since 2003.^3^, ^4^ Many countries have committed to an effort to decrease their production of greenhouse gases (GHG), primarily CO₂, to prevent an increase in temperature above 2°C, with the temperature of the pre‐industrial era used as the baseline. In this context, one point can be highlighted, namely the sustainable use of bioenergy.

The signatory countries of the Intergovernmental Panel on Climate Change (IPCC) have adopted different measures to contain the emissions of harmful gases; however, they all consider bioenergy as a source of renewable energy (RE). The European Union (EU), whose contribution to energy consumption (starting from 2458 PJ in 2005) will reach 4605 PJ in 2020, has adopted ambitious goals for using RE and bioenergy.^5^

The Conference of the Parties (COP21) of the United Nations Framework Convention on Climate Change (UNFCCC) resulted in an agreement to save the planet in the case that the expected scenario for extreme events and climate change is established. All the member countries committed to adopting measures to reduce GHG effects, and, in doing so, to mitigate the impacts of climate change. At the COP21, the participant countries presented their mitigating goals by their intended Nationally Determined Contributions (iNDCs). Each member country has established its iNDCs in the context of its national priorities, jurisdictions, and expertise, and these goals were endorsed in November 2016 at the COP22. For its iNDC, Brazil made a commitment to reduce its emissions of greenhouse gases by 37% below its 2005 levels by 2025, with a subsequent intention to reduce its emissions of greenhouse gases to 43% below the 2005 levels by 2030.^4^, ^6^, ^7^

Agricultural biomass residues are important and strategic inputs for bioenergy in the above context. Agricultural biomass residues were previously considered as an input of low aggregated value and were left behind in the field or burned; however, they are now seen as essential in a low‐carbon economy in several respects, including the composition of carbon in the soil, the mitigation of GHG effects, and the generation of renewable energy.^5^, ^8^, ^9^

Brazil has already made important sustainability advances in its use of agricultural biomass for energy generation. The production of ethanol, which may be used in pure form or in a mixture with gasoline as automobile fuel or for electric power generation (sugar mill cogeneration), provides two important examples.^10^, ^11^, ^12^ Additionally, it has been proposed that Brazil should adopt measures that are coherent with the temperature increase up to 2°C. Among these measures, expanding the use of renewable energy sources, except those derived from hydroelectricity, such as biomass, wind, and solar power energy, is highlighted. However, energy from hydroelectric power plants has also decreased due to the problems of water scarcity 13, 14, 15 and supply sources.^16^ In addition, the new hydroelectric power plants are located in the northern region, which is far from the region with the highest demand (in the southeast).^17^

Moreover, Brazil is engaged in implementing low‐carbon agriculture that focuses, among other things, on the use of biofuels and on increasing the alternative sources that biomass offers.^18^

Brazil is a country with great resources and varied agriculture, and it has enormous potential to engage in energy production using agricultural biomass residues.^19^ Many factors can define the major use of bioenergy. Among them is the relative geographic distribution of the sources of origin for agricultural biomass that could increase the diversity of the energy supply and contribute to improved energy security.^20^, ^21^, ^22^

Few studies have explored the availability of agricultural biomass residues as materials for exploitation as renewable energy sources in Brazil,^23^, ^24^ despite their widespread use around the world.

Therefore, key points need to be addressed, such as spatial aspects in the context of a low‐carbon economy. Agricultural biomass residues are spread over wide‐ranging territories. The Geographic Information System (GIS) is a powerful tool for assisting decision makers regarding agroenergy systems once the spatial variables are considered.^25^, ^26^, ^27^

Many authors have used spatial analyses to address the optimal energy use of agricultural residues,^5^, ^28^, ^29^, ^30^, ^31^ in which residues were exploited for potential energy generation. These studies considered spatial aspects and helped to create subsidies for the European community regarding public policy decision making. Other studies from around the world can also be mentioned; these studies include Voivontas et al^32^ in Greece, to estimate the biomass quantity potential for bioelectricity production; Sacchelli et al^33^ in Italy, who used a GIS model to quantify forestry biomass; Wakeyama and Ehara^34^ in Japan, who assessed the potential use of renewable energy in northern Tohoku; and Yousefi et al^35^ in Iran, who estimated renewable energy production from different sources of biomass.

In the United States of America (USA), the National Renewable Energy Laboratory (NREL) constantly performs evaluations on technological options for electric energy generation.^36^, ^37^ The evaluation of potential electrical energy generation is performed based on several sources, including those originating from agricultural residues and spatial analyses via GIS as a first step, without considering the cost. Voivontas et al^32^ studied plant capacities and the spatial distribution of residues, which are the primary parameters to consider regarding the location and size of the plant capacity.

The majority of studies consider spatial analyses as part of the decision support system at the municipality level and for the given electricity demand. However, in the energy sector, ethanol is currently the most important liquid biofuel,^38^ and performing an analysis via GIS can assist in managing both demands. Our proposal addresses a specific way to identify residues that is different from the usual analysis using satellite data, and it provides much greater accuracy in identifying the areas that have relevant residues.

Therefore, the objective of this study was to estimate the potential for electric energy generation and ethanol production by treating agricultural residues, namely sugarcane straw (SS) and eucalyptus forestry residues (EFR), while considering a spatial analysis.

This paper is organized as follows. Section 2 introduces the study area and the methods adopted to achieve the goal. Section 3 describes the results, and finally, Sections 4 and 5 present the discussion and conclusion, respectively.

## 2 MATERIAL AND METHODS

### 2.1 Study area

**Figure 1:**
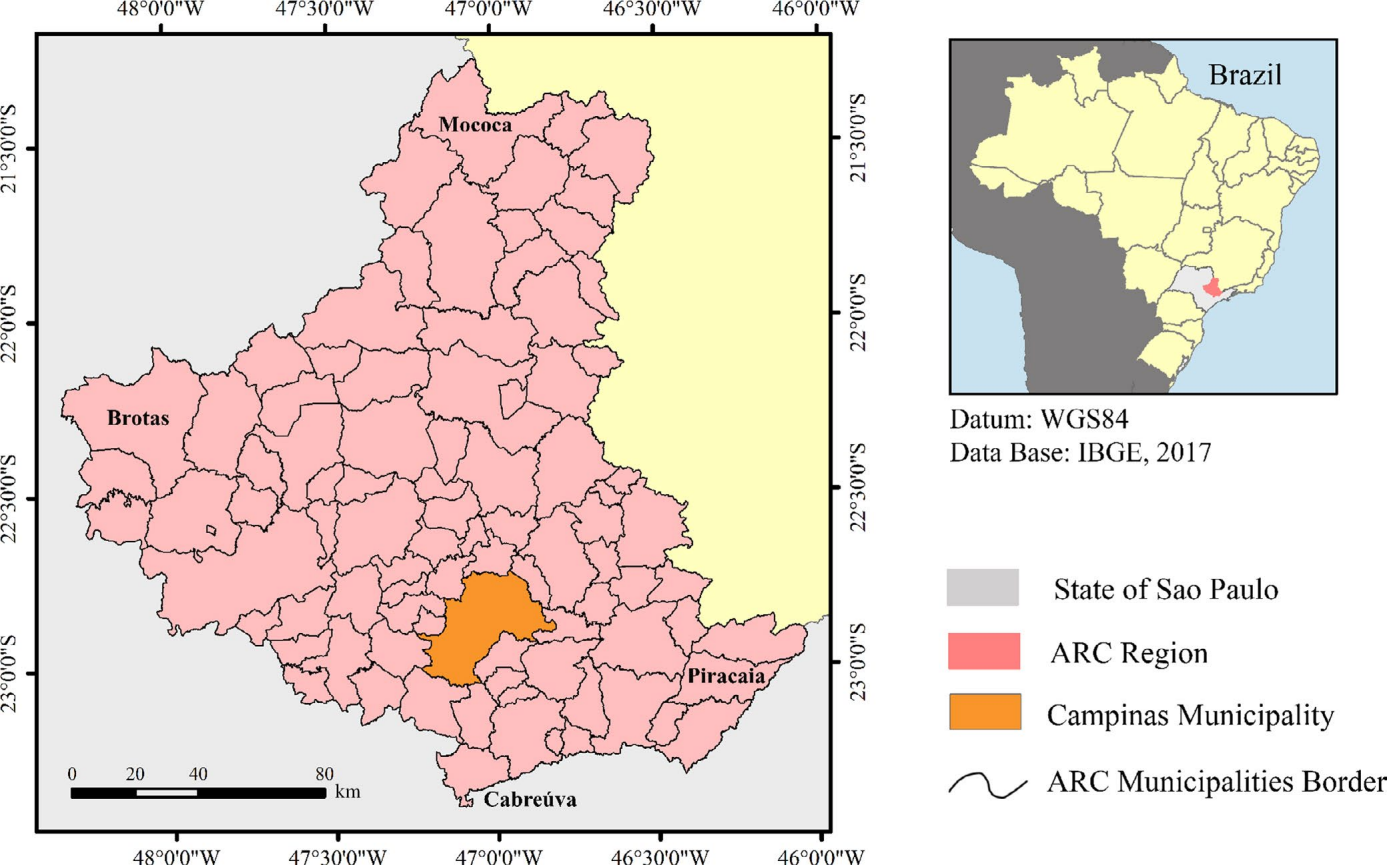
Geographic location of the ARC

The study area consisted of 90 municipalities (Figure 1), and it is known as the Administrative Region of Campinas (ARC). The ARC occupies an area of 27 079 km^2^ and represents 10.9% of the total territory in the state of São Paulo.^39^ This region has intensive agriculture that primarily consists of sugarcane to produce ethanol and sugar as well as forestry for the paper industry. The ARC is an intense energy consumer due to its industrial park and car fleet.

Part of the energy consumed by the ARC is provided by sugar mills that produce ethanol, which can be used in car engines directly or in mixture with gasoline. The other part is electric energy, which is provided by hydroelectric power plants or thermoelectric sources (fed by diesel).^39^ Determining the potential ethanol and electric energy production is the target of this work.

### 2.2 Material—Dataset

#### 2.2.1 Sugarcane and eucalyptus locations

**Figure 2:**
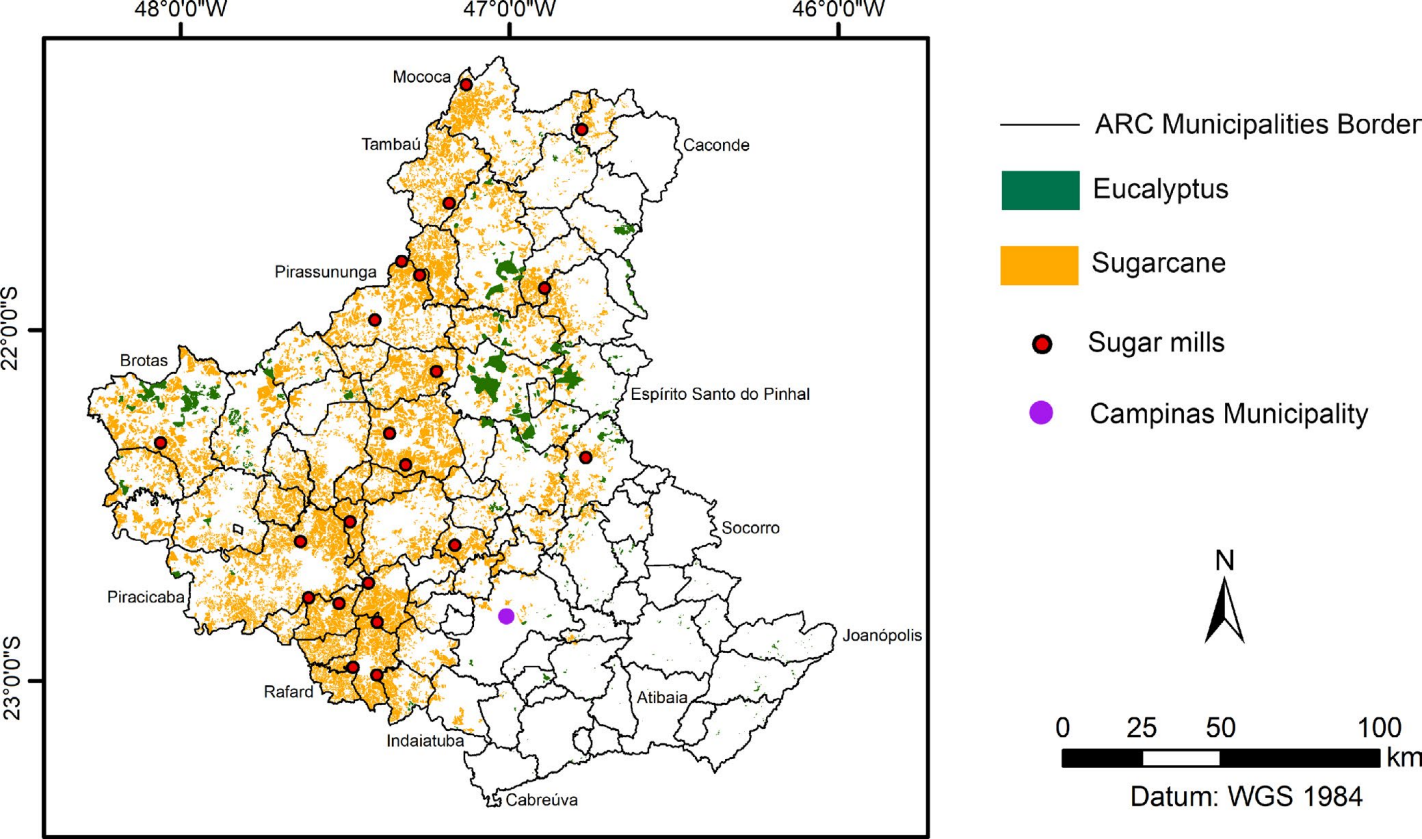
Sugarcane and eucalyptus production areas in the ARC

To identify and georeference the occupied area locations used for sugarcane production in the ARC (Figure 2), a satellite database obtained from the CanaSat Project was used along with information from the 2013/2014 harvest.^40^ This method identified the sugarcane areas by using medium‐resolution spatial images (30 m) from Landsat series satellites. The digital processing of the images was then supported by visual inspection. This area is very stable in terms of land cover. Because there is no more land available, the area occupied by sugarcane will not change. Thus, the sugarcane area from 2013/2014 has not changed, and this area was used in this study.

For the areas occupied with eucalyptus in the ARC (Figure 2), an information database was used from the Le Maire et al^41^; the authors mapped the eucalyptus plantations in Brazil from 2003 to 2010 using a binary classification method based on the MODIS (250 meters) Normalized Difference Vegetation Index (NDVI) time series.

##### Electrical energy consumption by municipality

The information in Table 1 was extracted from the Annual Energetic Report by municipality for the state of São Paulo in 2016^42^ and for the energy balance of the state of São Paulo.^42^ The annual report is based on information from 2015, and it was prepared by the State Secretary for Energy and Mining. The annual report includes consolidated data about the primary energy consumption by the 645 municipalities in the state of São Paulo.

**Table 1:** Consumption of electricity by municipalities in the ARC, in descending order

Item	Municipalities	Tera Joule (TJ year^−1^)	Item	Municipalities	Tera Joule (TJ year^−1^)
1	Campinas	12 002.22	46	Socorro	288.50
2	Piracicaba	7499.20	47	Espírito Santo do Pinhal	277.88
3	Jundiaí	7192.26	48	Holambra	277.63
4	Limeira	4793.47	49	Elias Fausto	276.52
5	Americana	4479.19	50	Morungaba	273.56
6	Paulínia	3709.37	51	Engenheiro Coelho	270.58
7	Sumaré	3394.40	52	Casa Branca	262.84
8	Rio Claro	3076.60	53	Conchal	238.43
9	Indaiatuba	3061.98	54	São Pedro	236.48
10	Mogi Guaçú	2570.18	55	Serra Negra	223.74
11	Sta Bárbara d'Oeste	2385.97	56	Iracemápolis	188.68
12	Hortolândia	2004.01	57	Santo Antônio de Posse	179.21
13	Bragança Paulista	1891.80	58	Brotas	177.48
14	Valinhos	1691.39	59	Águas de Lindóia	170.60
15	Araras	1593.50	60	Piracaia	168.30
16	Sta Gertrudes	1592.82	61	Santa Cruz das Palmeiras	153.18
17	Mogi‐Mirim	1557.76	62	Itirapina	143.57
18	Vinhedo	1543.86	63	Joanópolis	140.76
19	Atibaia	1507.86	64	Santa Cruz da Conceição	119.41
20	Itatiba	1359.94	65	Nazaré Paulista	116.06
21	Jaguariúna	1322.24	66	Corumbataí	116.06
22	Amparo	1319.36	67	Charqueada	112.86
23	São João da Boa Vista	1271.52	68	Ipeúna	110.56
24	Nova Odessa	1256.54	69	Lindóia	98.28
25	Cordeirópolis	1095.98	70	Rafard	88.74
26	Itupeva	1056.20	71	Pinhalzinho	87.66
27	Várzea Paulista	1041.77	72	Saltinho	78.37
28	Louveira	1008.65	73	Caconde	76.86
29	Cabreúva	941.15	74	Tapiratiba	74.95
30	Leme	829.62	75	Itobi	68.54
31	Itapira	825.12	76	Divinolândia	65.02
32	Capivari	758.81	77	Estiva Gerbi	62.78
33	Pedreira	715.86	78	São Sebastião da Grama	60.30
34	Pirassununga	647.24	79	Águas da Prata	57.20
35	São José do Rio Pardo	593.64	80	Torrinha	56.45
36	Mococa	592.16	81	Monte Alegre do Sul	53.14
37	Monte Mor	578.23	82	Águas de São Pedro	50.80
38	Cosmópolis	412.96	83	Vargem	49.97
39	Vargem Grande do Sul	356.15	84	Santa Maria Da Serra	45.79
40	Jarinu	348.16	85	Mombuca	39.20
41	Rio das Pedras	342.00	86	Santo Antônio do Jardim	36.07
42	Artur Nogueira	334.94	87	Tuiuti	35.86
43	Bom Jesus dos Perdões	309.06	88	Analândia	35.68
44	Aguaí	298.87	89	Pedra Bela	31.10
45	Tambaú	298.48	90	Campo Limpo Paulista	22.46
Total			93 260.63 TJ year^−1^		

Table 1 shows information about the electricity consumption by the municipalities in the ARC. The total consumption was almost 93 000 terajoules (TJ). These data will be compared with those calculated from the available production of biomass residues, and an evaluation of the demands and consumption and the deficits and surplus in the region is provided.

#### 2.2.2 Ethanol Consumption in the ARC by municipality

The information included in Table 2 was extracted from the State of São Paulo Energy‐per‐Municipality Yearbook of 2016^42^ and the Energy Balance of the State of São Paulo.^43^ Table 2 provides information about ethanol consumption for each municipality in the ARC. The total consumption was 1850 megaliters (ML).

**Table 2:** Total consumption of bioethanol per municipality, in descending order

Item	Municipalities	Ethanol Consumption (ML)	Item	Municipalities	Ethanol Consumption (ML)
1	Campinas	325.42	46	Vargem Grande do Sul	6.01
2	Jundiaí	140.51	47	Cabreúva	5.74
3	Piracicaba	131.34	48	Águas de Lindóia	5.52
4	Limeira	106.70	49	Aguaí	5.31
5	Americana	83.80	50	Rio das Pedras	5.27
6	Sumaré	64.55	51	Serra Negra	5.23
7	Indaiatuba	57.91	52	Divinolândia	5.06
8	Santa Bárbara d'Oeste	54.78	53	Tambaú	4.98
9	Valinhos	49.89	54	Conchal	4.95
10	Hortolândia	47.84	55	Iracemápolis	4.37
11	Rio Claro	44.61	56	Piracaia	4.10
12	Atibaia	41.93	57	Pinhalzinho	4.01
13	Mogi‐Mirim	40.63	558	Jarinu	3.87
14	Mogi Guaçú	39.00	59	Tapiratiba	3.60
15	Bragança Paulista	38.38	60	Holambra	3.45
16	Paulínia	33.90	61	Lindóia	3.45
17	Itatiba	30.62	62	Bom Jesus dos Perdões	3.43
18	Araras	30.22	63	Vargem	3.41
19	São João da Boa Vista	25.71	64	Águas de São Pedro	3.15
20	Vinhedo	23.58	65	Santa Cruz da Conceição	3.14
21	Leme	23.29	66	Caconde	3.07
22	Pirassununga	22.54	67	Itirapina	3.01
23	Mococa	21.87	68	Morungaba	2.82
24	Nova Odessa	20.84	69	Engenheiro Coelho	2.63
25	São José do Rio Pardo	17.64	70	Santa Gertrudes	2.32
26	Várzea Paulista	16.35	71	Torrinha	2.07
27	Jaguariúna	15.75	72	Elias Fausto	2.06
28	Amparo	15.26	73	Joanópolis	1.97
29	Itapira	15.01	74	Nazaré Paulista	1.72
30	Monte Mor	11.91	75	São Sebastião da Grama	1.62
31	Itupeva	11.50	76	Charqueada	1.62
32	Capivari	10.68	77	Monte Alegre do Sul	1.58
33	Campo Limpo Paulista	10.12	78	Saltinho	1.55
34	Espírito Santo do Pinhal	9.80	79	Itobi	1.45
35	Santo Antônio de Posse	9.66	80	Estiva Gerbi	1.25
36	Artur Nogueira	9.63	81	Águas Da Prata	1.23
37	Cosmópolis	9.22	82	Santo Antônio do Jardim	1.22
38	Louveira	9.17	83	Mombuca	1.14
39	São Pedro	9.04	84	Rafard	0.98
40	Socorro	8.37	85	Analândia	0.98
41	Pedreira	7.84	86	Ipeúna	0.97
42	Cordeirópolis	7.47	87	Santa Maria da Serra	0.97
43	Santa Cruz das Palmeiras	6.98	88	Pedra Bela	0.96
44	Casa Branca	6.74	89	Corumbataí	0.55
45	Brotas	6.01	90	Tuiuti	0.43
Total			1852.19 (ML)		

### 2.3 Methods

#### 2.3.1 Estimated amounts of agricultural residues

In this study, the estimated agricultural residues are calculated by considering one process for the same power plant as separated by each type, as follows:

##### Estimated residue availability from sugarcane

Sugarcane is the most cultivated crop in the ARC. Sugarcane residues that come from agricultural production can be used as raw material to produce electrical power and bioproducts. Despite Brazil's demonstrably positive conditions for developing second‐generation ethanol derived from sugarcane biomass,^44^ we only consider first‐generation ethanol production in this study.

Specifically, in relation to sugarcane straw (SS is dry leaves, green leaves, and tops), according to Menandro et al,^45^ the performance of dry mass SS in the field is 14 Mg.ha^‐1^. From this total mass, the same authors suggested that 60% (8.4 Mg ha^‐1^) of the dry leaves could be exploited to guarantee agronomic sustainability. The availability of the residues was then estimated using those parameters along with the sugarcane area obtained by CanaSat.

##### Estimate of Eucalyptus Forestry Residue availability (EFR)

Eucalyptus plantations are present in approximately 40% of the municipalities in the ARC. Mogi Guaçu (MG), Espírito Santo do Pinhal (ESP), Casa Branca (CB), and Brotas (BRO) are the leading municipalities for producing wood that originates from eucalyptus. Because of wood exploitation, forestry residues are important sources of the lignocellulosic biomass used for energy.^46^, ^47^

The amount of forestry residues, which basically include bark, leaves, and stalks in designated areas for eucalyptus forestry use, varies from 10 to 70 Mg.ha^‐1^, according to Wrobel‐Tobiszewska et al^48^ In this study, only the bark and stalk dry basis was considered, because these residues are present in higher amounts. According to Foelkel,^49^ in Brazil, the availability of eucalyptus residues (such as bark and stalks) in the field is 30 Mg ha^‐1^, which is within the range presented in Wrobel‐Tobiszewska et al^48^ Therefore, the value of 30 Mg ha^‐1^ was used to calculate the amount of available residues by considering the total eucalyptus area from satellite data.

##### Residue availability

We are assuming that the residues will be available during the harvest time for sugarcane and eucalyptus. The sugarcane harvest begins in April and ends in December. The eucalyptus harvest was considered throughout the year. Therefore, the total amount of residues was divided into 9 months for sugarcane, for 598 × 10^3^ Mg per month, and 12 months for eucalyptus, for 79 × 10^3^ Mg per month.

#### 2.3.2 GIS‐based model

**Figure 3:**
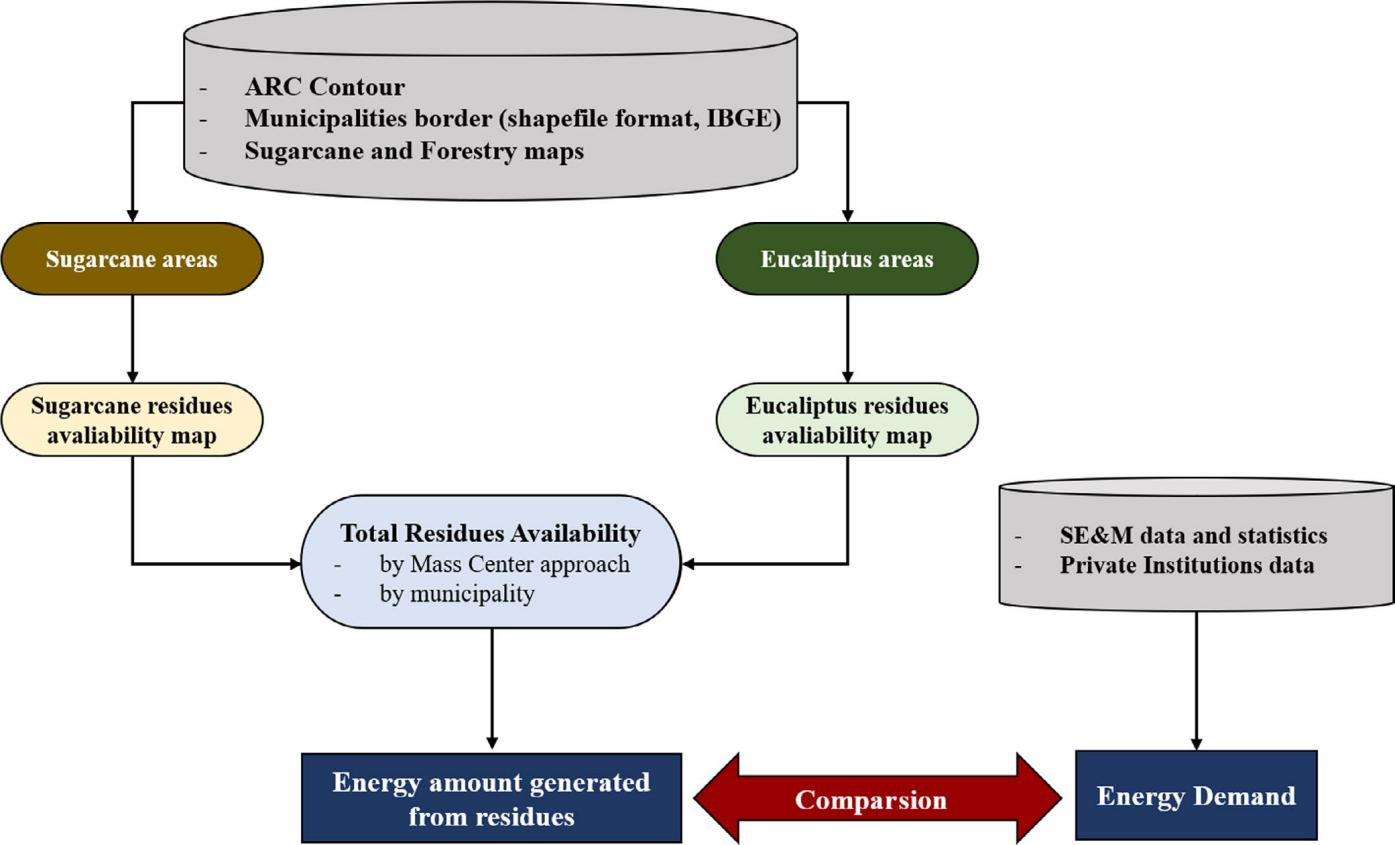
Flowchart of the analysis steps

For the good organization of each identified variable, sugarcane and forestry area maps as well as municipality borders were added to the GIS system as a layer. To estimate the residue (straw from sugarcane and residues from eucalyptus) amounts per municipality and per mass center buffer approach, the layer areas and borders were overlaid. A calculation of the residue amount and energy resources, such as for the electric energy (EE) and ethanol, was performed. The last step considered the evaluation comparison between the demand and consumption for EE and ethanol (Figure 3).

#### 2.3.3 Estimates of potential energy generation (PEG)

Evaluating the technical potential of decentralized energy production by SS and EFR depends on having a consistent database, which begins with the quantification of their availability. The agronomic requisites for soil conservation and the EFR and SS gravimetric compositions are essential variables for energy exploitation studies.

Notably, the heterogeneity of SS and EFRs makes it difficult to select a technological route for energy exploitation, to ensure compatibility with the evaluated residues. This characteristic provides multiple possibilities for chemical technologies that can be used to exploit a specific residue. The primary interest in this study was to evaluate the energetic potential from the lower calorific values (LCVs) individually for two agricultural residues without emphasizing the relevance of one technology in relation to the other. Then, the technological biochemical route was chosen to estimate the energy from SS and EFRs. This process is based on the enzymatic decomposition of organic matter by microbes via codigestion to produce biogas and subsequently generate electrical energy.^50^ Biochemical conversion processes are recommended for residues with a high percentage of biodegradable organic material and high‐humidity content.

##### From SS

The technical potential of generating energy from SS was estimated by considering the technological route of anaerobic digestion via codigestion with vinasse. Vinasse was identified because there is a series of sugarcane and ethanol mills around the ARC (Figure 2). According to Viana,^51^ the average monthly LCV of SS (June to October) is 17 584.52 MJ Mg^‐1^. To estimate the electric energy generation, the following energetic indicator was used, and it considers the availability of SS and LCV.

##### From EFR

The inventory and definition of EFR represent the study basis for evaluating the EFR potential for energy generation. During industrial wood processing from tree seeding to the tree harvest, a high percentage of organic matter is usually generated. Common sense dictates that residues are the remains that occur from harvest processing, and they are not incorporated into the final product.^52^, ^53^

The LCVs are very similar among the bark and stalks.^49^, ^54^, ^55^ Thus, in this study, an average value of 17 165.84 MJ Mg^‐1^ was used for the dry base, according to Foelkel.^49^ The final PGE considered the total eucalyptus residue availability as well as the average LCV.

#### 2.3.4 Estimates of the potential ethanol production

##### From SS

The use of biomass as a raw material for new products opens up the possibility of producing energy and biofuels as bioethanol. The amount of ethanol that can be produced can be assessed by multiplying the sugarcane straw availability by the indicator, which is 287 L Mg^‐1^ of straw.^56^

Sugarcane straw, which is the aerial part of the plant (dry and green leaves and tops) except for the industrially treated stalks, is basically made up of cellulose (40%), hemicelluloses (30%) and lignin (25%).^55^ Nevertheless, according to Santos et al, studies performed with *in natura* sugarcane straw have displayed a composition of 38% cellulose, 29% hemicelluloses and 24% lignin. The ash content is typically two to four times higher compared with sugarcane bagasse. This amount can vary depending on the material collection site, weather conditions, vegetative development stage, and cultivar. An understanding of the structural complexity of the lignocellulosic materials requires knowledge of the physicochemical properties of each of their cell wall components to determine the exact energy potential.

##### From EFR

In terms of chemical composition, the plant cell wall of eucalyptus is made of cellulose, hemicellulose, and lignin. Many studies related to the manipulation of lignin biosynthesis have been conducted.^57^, ^58^ There is strong interest in this field due to the possibility of producing plants that are more appropriate for the delignification processes used to produce cellulose as well as the new industry of converting biomass to turn lignified biomass into bioethanol.^59^

Producing bioethanol from residual lignocellulose has great environmental appeal if the emissions of CO_2_ into the atmosphere are compensated for by the absorption of the gas during new plant biomass development. Brazil has special conditions if we consider the lignocellulosic residues from the forestry sector, because the residual biomasses are available in a reasonably clean form and in large amounts.^60^

Bragatto^61^ and Matsushita et al^62^ showed the technical potential of bioethanol production from EFRs. In their studies, evaluations were performed on the residue chemical compositions, total soluble carbohydrate extraction mechanisms, acid and alkaline pretreatment processes, enzymatic hydrolysis, and a comparative analysis with sugarcane bagasse. The ethanol production process from soluble sugars is considered 1G fuel, and it does not involve breaking the cell wall. The bioethanol production per hectare is 1600 liters per hectare, according to 1G routes.^61^

The ethanol consumption data (Table 2) were compared with the ethanol amounts calculated from the available residue production with an evaluation of the demands and consumption and, thus, the deficits and surplus figures for the region.

#### 2.3.5 Spatial distribution of crop residue areas based on a mass center approach

The municipalities were grouped according to a spatial clustering standard on the residue availability for sugarcane and eucalyptus. For this reason, the methodology was based on the availability of the total residues in the ARC per real occupied area as follows:

1. Identify the major producers of residues based on the available statistical and georeferenced information;
2. Characterize the possible spatial structure of those municipalities in terms of residue availability.

An analysis of the potential residue production (sugarcane and forestry) in the ARC was performed by median center (mass center) approach. This method is an iterative procedure first used by Kuhn and Kuenne (1962)^63^ and refined by Burt and Barber.^64^ At each step *(t)* in the algorithm, a candidate median center is found *(X^t^, Y^t^)* and then optimized until it represents the location that minimizes the Euclidean distance *d* to all the features *(i)* in the dataset (Equation 1).

(1) $$d_{i}^{t}=\sqrt{{\left(x_{i}-x^{t}\right)}^{2}-{\left(y_{i}-y^{t}\right)}^{2}}.$$

This approach allows the user to reach the best results while considering the true location of the planted areas, whether eucalyptus or sugarcane, instead of using aggregated values, such as the statistics from the municipalities.

## 3 RESULTS

**Figure 4:**
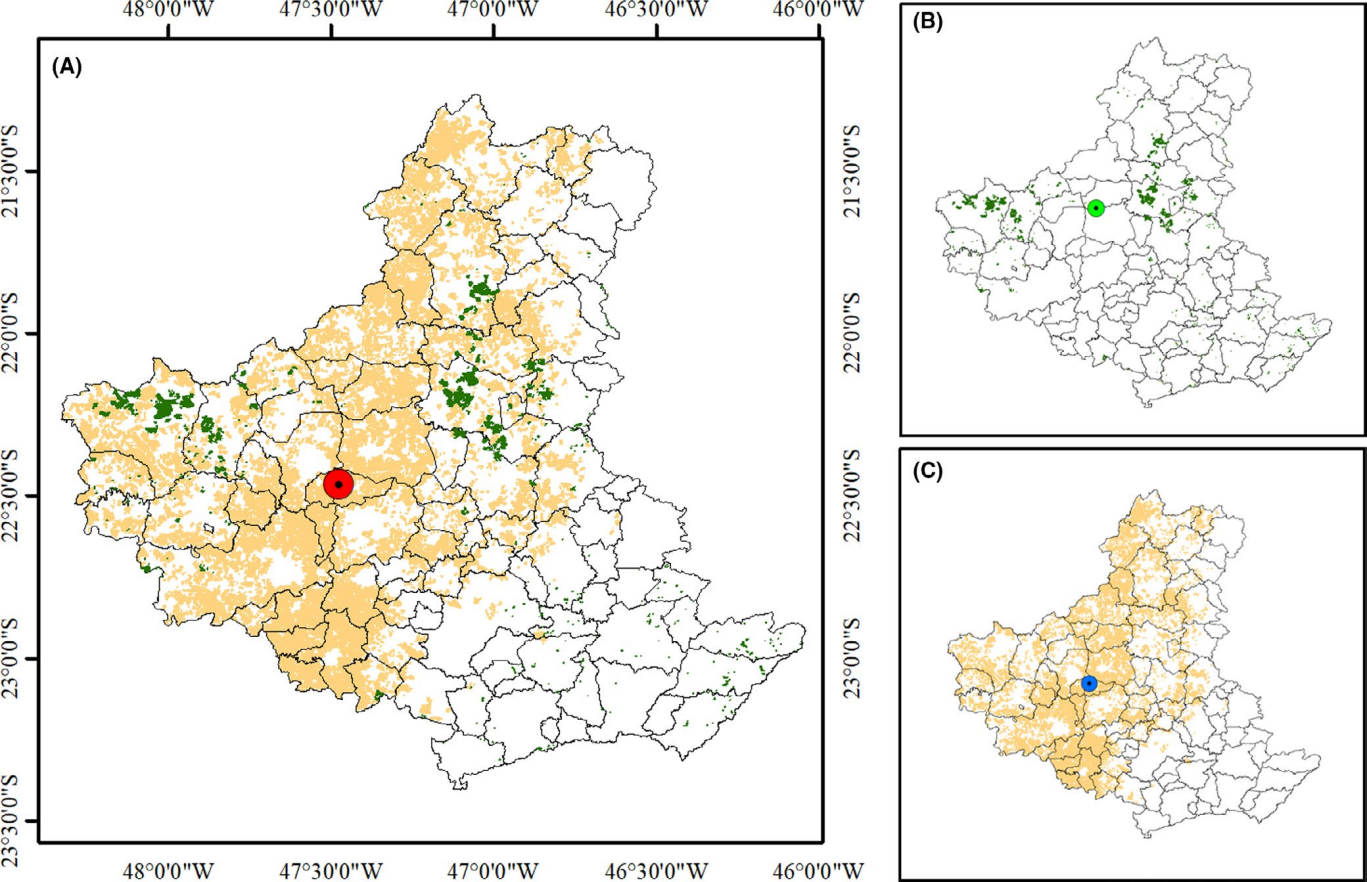
Available amounts of the related residues. Distribution of sugarcane and eucalyptus areas (A) and center of mass (CM_T_) together, CM_E_ eucalyptus (B), and CM_S_ for sugarcane (C)

Figure 4 shows the distribution of residues per ARC municipality for sugarcane and forestry according to the described methodology. The sugarcane residues were more available in the central‐western to northern regions. Regarding eucalyptus, however, the residues were distributed in the central‐eastern to northern regions. The map shows a clear overlap of the residue availability, which operationally aids their exploitation.

The points in red (Figure 4A), green (Figure 4B) and blue (Figure 4C) are the centers of mass (CM) related to both crops/plantations (sugarcane‐S and eucalyptus‐E), sugarcane, and eucalyptus (T), respectively. This approach allows the user to identify the best place where a residue processing mill could be placed.

From the CM_T_, in red, buffers were generated to analyze the data. It was considered only a CM because the difference from the sugarcane CM_S_ and eucalyptus CM_E_ was the minimum (± 23 km).

**Figure 5:**
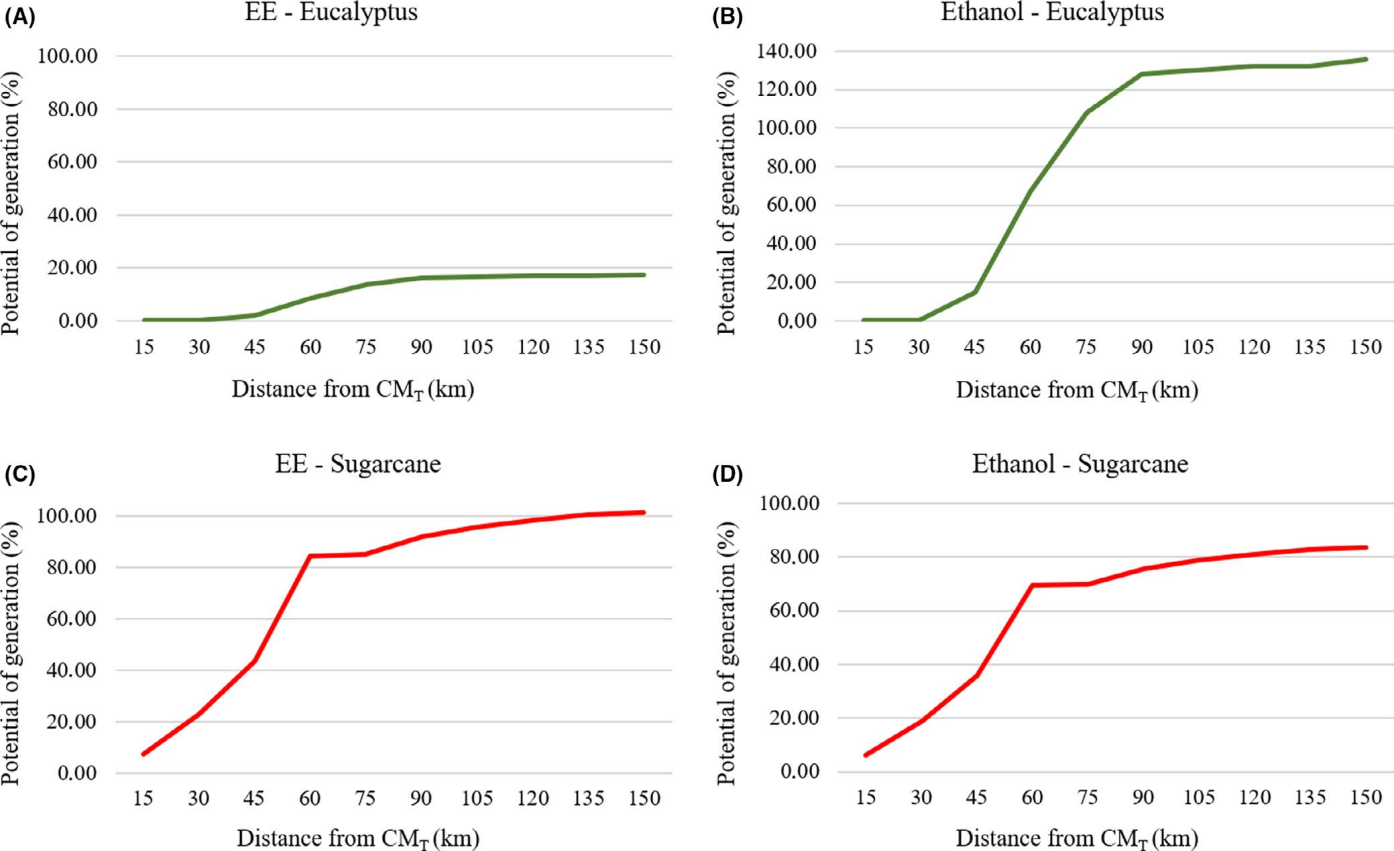
Potential generation of EE and ethanol, in kilometers from the CM_T_, as based on the percentage consumption by the ARC

Sugarcane residues are available from April until December. However, they can provide 100% of the EE but only up to 85% of the ethanol needs of the ARC, when considering a buffer of 90 km (Figure 5C, 5). However, despite providing only 18% of the EE for ARC needs (buffer 75 km; Figure 5A), the eucalyptus residues can supply energy during the entire year, including time outside of the sugarcane harvest period. In terms of ethanol (buffer 75 km; Figure 5B), the production only supplies the alcohol needed by the ARC. During the other part of the year (April to December), the eucalyptus can be added, increasing the potential energy supply.

Tables 3 and 4 discriminate between the production area and net residue availability for sugarcane and eucalyptus per municipality, respectively. The following analysis will consider the net residue availability around the buffer built from CM_T_, that is, using sugarcane and eucalyptus, as mentioned previously.

**Table 3:** Production area and availability of net residues per municipality, for sugarcane

Municipalities	Production area (ha)	Net waste (Mg)
Piracicaba (PIR)	59 906.0	503 210.4
Araras (ARA)	36 053.0	302 845.2
Brotas (BRO)	32 425.4	272 373.9
Pirassununga (PRG)	30 408.3	255 430.0
Capivari (CAP)	26 765.7	224 832.6
Santa Bárbara d'Oeste (SBO)	24 599.8	206 639.0
Mococa (MOC)	22 641.3	190 187.5
Tambaú (TAM)	20 959.7	176 061.8
Leme (LEM)	19 890.8	167 083.2
Rio das Pedras (RP)	19 812.0	166 421.3
Santa Cruz das Palmeiras (SCP)	18 352.4	154 160.4
Limeira (LIM)	17 612.5	147 945.5
Rio Claro (RC)	16 292.0	136 852.9
Aguaí (AGU)	14 121.0	118 616.9
Mombuca (MOM)	11 776.9	98 926.1
Torrinha (TOR)	11 289.1	94 829.1
Iracemápolis (IRA)	11 044.8	92 777.0
Charqueada (CHA)	10 358.1	87 008.1
Cordeirópolis (COR)	10 345.6	86 903.5
Elias Fausto (EF)	9444.4	79 333.6
Rafard (RAF)	9435.3	79 257.2
Monte Mor (MM)	9261.8	77 799.6
Analândia (ANL)	8636.6	72 547.5
Vargem Grande do Sul (VGS)	7768.5	65 256.0
Cosmópolis (COS)	7576.1	63 639.7
Ipeúna (IPE)	7423.45	62 357.0
Santa Gertrudes (SG)	7170.50	60 232.4
Santa Maria da Serra (SMS)	6398.60	53 748.8
Santa Cruz da Conceição (SCC)	5686.10	47 763.2
Sumaré (SUM)	4566.00	38 354.8
Saltinho (SAL)	4404.50	36 998.1
Nova Odessa (NO)	3938.30	33 081.7
Americana (AME)	3731.60	31 345.5
Santo Antônio de Posse (SAP)	3665.30	30 789.0
Paulínia (PAU)	3522.70	29 591.3
Jaguariúna (JAG)	3443.80	28 928.3
Engenheiro Coelho (EC)	2844.60	23 894.7

**Table 4:** Production area and availability of net residues per municipality, for eucalyptus

Municipalities	Production area (ha)	Net waste (Mg)
Mogi Guaçú (MG)	12 742.2	382 266.1
Brotas (BRO)	9664.4	289 932.1
Casa Branca (CB)	5956.3	178 691.3
Espírito Santo do Pinhal (ESP)	4594.3	137 829.0
Itirapina (ITI)	3424.4	102 733.6
Aguaí (AGU)	2260.9	67 827.7
Itapira (ITA)	1886.4	56 593.9
São Sebastião da Grama (SSG)	1545.7	46 373.5
Torrinha (TOR)	989.4	29 682.8
Águas da Prata (AP)	975.5	29 265.2
Analândia (ANA)	926.4	27 794.2
Conchal (CON)	919.0	27 570.8
Corumbataí (COB)	738.4	22 154.1
Artur Nogueira (AN)	593.2	17 796.0
Estiva Gerbi (EG)	415.1	12 454.2
Santo Antônio do Jardim (SAJ)	408.9	12 267.6
Vinhedo (VIN)	304.4	9133.5
Monte Alegre do Sul (MAS)	296.9	8908.8

**Figure 6:**
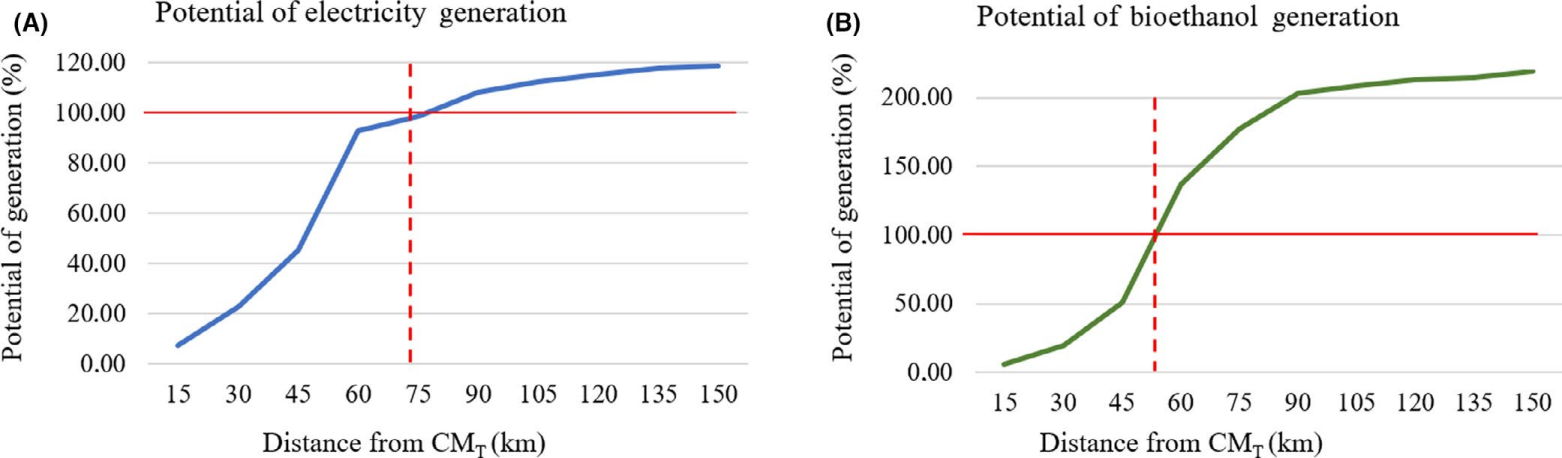
Cut basis, in kilometers from the CM_T_, for generating energy

Using a buffer of 75 km to generate EE (107 658 TJ year^‐1^) (Figure 6A) as well as a buffer of 50 km for ethanol consumption (1852 ML year^‐1^) (Figure 6B) would be enough to meet the needs of the ARC.

**Figure 7:**
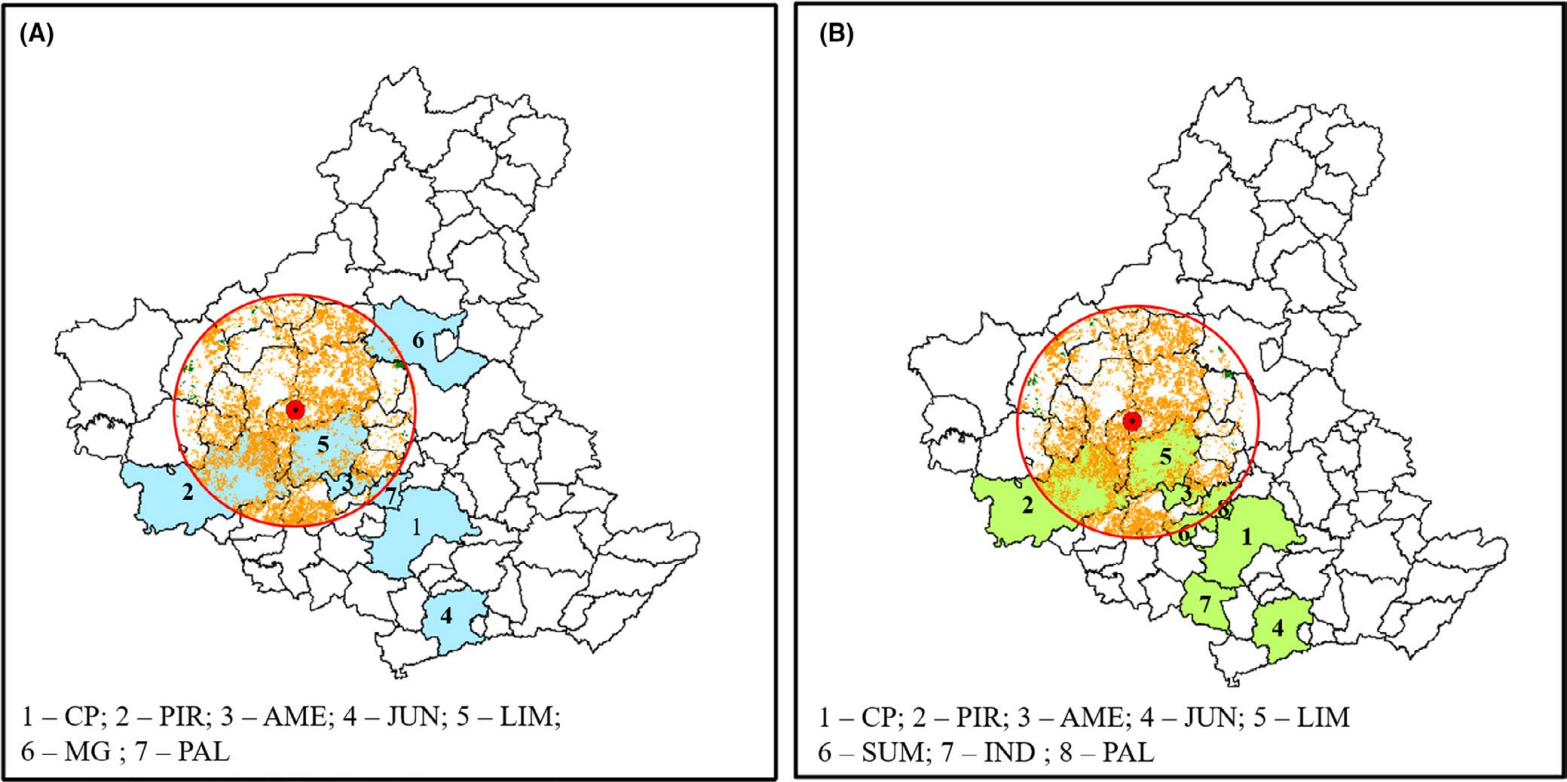
Residue availability in a buffer of 45 km from the CM_T_

**Figure 8:**
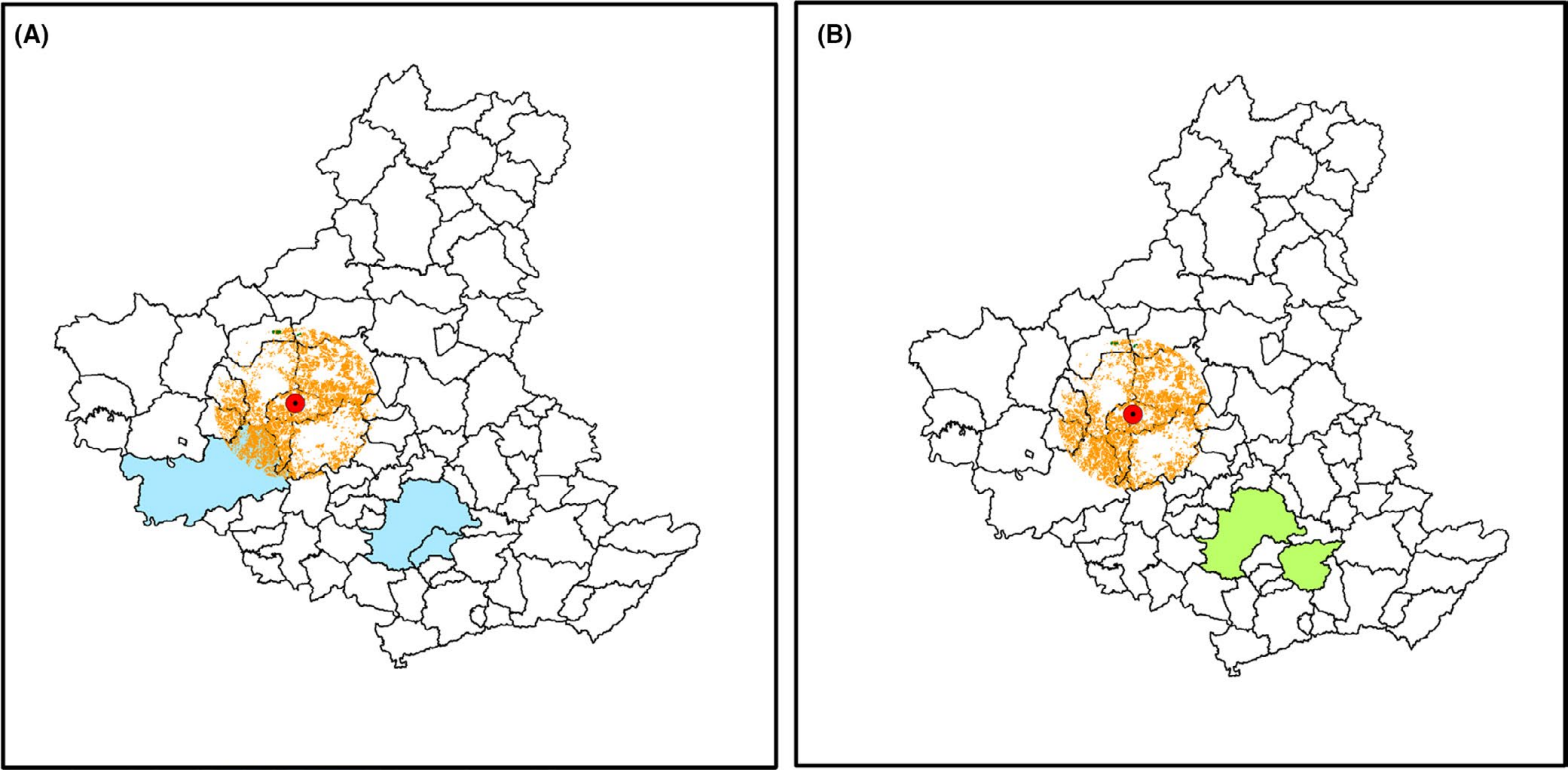
Residue availability with a buffer of 30 km from the CM_T_

Some scenarios could be configured:

1. For a buffer of 45 km, the residue availability will provide EE to the seven highest consuming municipalities in the ARC (Figure 7A), while in the same buffer zone, the ethanol consumption needs can be met for eight municipalities (Figure 7B), and six of those municipalities are the same as the highest consumers of both ethanol and EE.
2. For a buffer of 30 km, the residue availability can meet the needs of three municipalities, which include the biggest consumers in EE (Figure 8A), or two municipalities regarding the ethanol consumption needs (Figure 8B).

Considering the 10 municipalities that are the highest consumers of energy, 7 are the same regarding EE and ethanol consumption (Campinas (CP), Piracicaba (PIR), Jundiaí (JUN), Limeira (LIM), Americana (AME), Sumaré (SUM), and Indaiatuba (IND)). For EE, the municipalities of Paulínia (PAU), Rio Claro (RC), and Mogi Guaçu (MG) stand out in these groups because they have the highest human development index (HDI), with an average of 0.791, compared with the other three municipalities of Santa Bárbara d’Oeste (SBO), Valinhos (VAL), and Hortolândia (HOR), which have an average of 0.785. However, regarding ethanol consumption, the municipalities of SBO, VAL, and HOR have 15% more cars in relation to the cited municipalities of PAU, RC, and MG, as shown in Tables 5 and 6.

**Table 5:** The 10 municipalities with the highest consumption of EE

Ranking	Municipalities	EE consumptions (TJ year^−1^)	EE index	HDI
1	Campinas (CP)	12002.25	1.000	0.805
2	Piracicaba (PIR)	7499.20	0.625	0.785
3	Jundiaí (JUN)	7192.28	0.599	0.822
4	Limeira (LIM)	4793.47	0.399	0.775
5	Americana (AME)	4479.22	0.373	0.811
6	Paulínia (PAU)	3709.39	0.309	0.795
7	Sumaré (SUM)	3394.42	0.283	0.762
8	Rio Claro (RC)	3076.63	0.256	0.803
9	Indaiatuba (IND)	3061.99	0.255	0.788
10	Mogi Guaçú (MG)	2570.19	0.214	0.774

**Table 6:** The 10 municipalities with the highest consumptions of ethanol

Ranking	Municipalities	Ethanol (GL year^−1^)	Ethanol index	Number of cars^a^
1	Campinas	325 415.50	1.000	589 772
2	Jundiaí	140 514.18	0.432	201 842
3	Piracicaba	131 344.60	0.404	174 610
4	Limeira	106 700.06	0.328	122 669
5	Americana	83 800.00	0.258	106 901
6	Sumaré	64 546.62	0.198	101 118
7	Indaiatuba	57 908.90	0.178	102 786
8	Santa Bárbara d'Oeste	54 781.26	0.168	82 067
9	Valinhos	49 893.90	0.153	61 240
10	Hortolândia	47 844.00	0.147	70 207

a number of cars running on ethanol

### 3.1 Consumption and demand balance: electricity analysis by municipality

**Figure 9:**
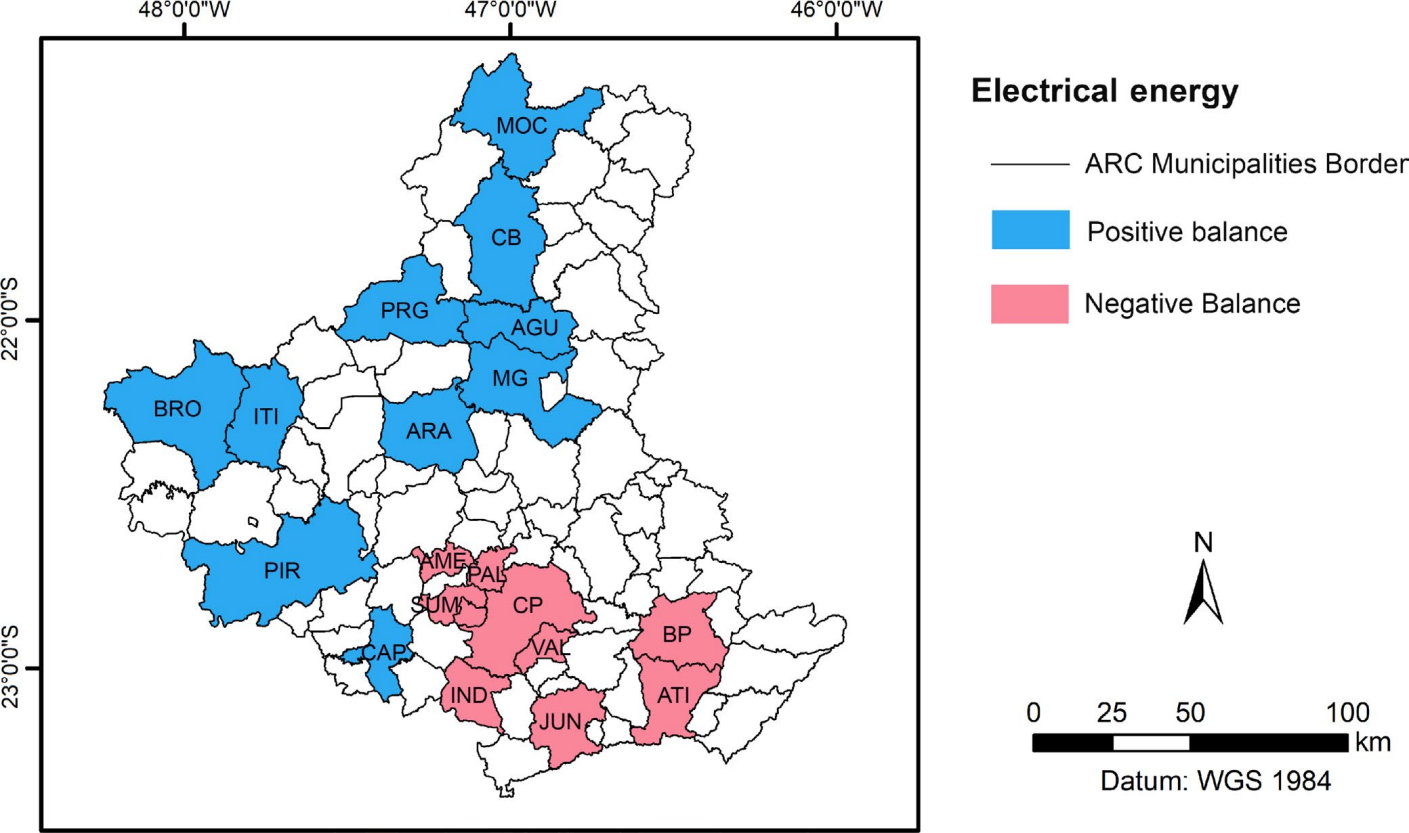
Locations of the municipalities that have negative and positive balances

By comparing the information in Table 1 with that in Table 7 for the study area as a whole, the consumption of EE was 93 260.63 TJ year^‐1^, whereas the EE generated from the residues could reach 170 382.54 TJ year^‐1^. The difference between these numbers is almost 55%; that is, the generated EE supplies all the consumption. Figure 9 shows the spatial distribution of the municipalities that have a positive balance (the generation of EE is higher than the consumption) and the municipalities in which the balance is negative (the generation of EE is lower than the consumption).

**Table 7:** Amount of EE generated from the total of net residue available from eucalyptus and sugarcane

RAC municipalities	Total residues (Mg)	PGE (TJ year^−1^)	RAC municipalities	Total residues (Mg)	PGE (TJ year^−1^)
Brotas	562 306.05	13 609.38	Saltinho	36 998.11	895.46
Piracicaba	521 652.59	12 625.45	Campinas	34 157.95	826.72
Mogi Guaçú	477 295.21	11 551.88	Sto Antônio de Posse	33 324.31	806.54
Casa Branca	337 256.30	8162.55	Nova Odessa	33 081.75	800.67
Araras	302 845.24	7329.70	Americana	31 345.54	758.65
Pirassununga	255 430.01	6182.12	Águas da Prata	30 556.55	739.55
Capivari	224 832.67	5441.58	Paulínia	29 591.35	716.19
Santa Bárbara d'Oeste	206 639.03	5001.24	Jaguariúna	28 928.38	700.15
Mococa	201 276.34	4871.45	Indaiatuba	27 412.81	663.47
Itirapina	198 370.02	4801.11	Engenheiro Coelho	23 894.74	578.32
Aguaí	186 444.73	4512.48	Estiva Gerbi	22 838.29	552.75
Tambaú	179 533.78	4345.22	Itobi	14 648.55	354.54
Leme	172 763.59	4181.36	Sto Antônio do Jardim	12 267.67	296.91
Rio das Pedras	166 421.38	4027.86	Joanópolis	10 410.07	251.95
Espírito Sto do Pinhal	158 050.10	3825.25	Holambra	9736.03	235.64
Santa Cruz das Palmeiras	154 160.47	3731.12	Piracaia	9352.15	226.35
Limeira	147 945.56	3580.70	Vinhedo	9133.55	221.06
Rio Claro	136 852.91	3312.22	Monte Alegre do Sul	8908.87	215.62
São Pedro	133 614.18	3233.84	Morungaba	7086.79	171.52
Torrinha	124 512.00	3013.54	Serra Negra	6928.02	167.68
Itapira	121 473.85	2940.01	Bragança Paulista	6246.83	151.19
Analândia	100 341.72	2428.55	Caconde	4995.12	120.90
Mombuca	98 926.14	2394.29	Nazaré Paulista	4454.72	107.82
Iracemápolis	92 777.06	2245.46	Itatiba	4103.47	99.32
Moji Mirim	89 081.50	2156.02	Pedreira	3616.32	87.53
Charqueada	87 188.28	2110.20	Jundiaí	3137.51	75.94
Elias Fausto	87 047.60	2106.80	Pedra Bela	2511.27	60.78
Cordeirópolis	86 903.54	2103.31	Itupeva	2383.44	57.69
São João da Boa Vista	81 846.18	1980.91	Jarinu	1961.34	47.47
Rafard	79 257.26	1918.25	Divinolândia	1107.46	26.80
Monte Mor	77 799.64	1882.97	Cabreúva	963.92	23.33
Vargem Grande do Sul	67 880.70	1642.90	Socorro	959.22	23.22
Corumbataí	65 406.55	1583.02	Vargem	860.41	20.82
Ipeúna	64 264.32	1555.38	Valinhos	683.14	16.53
Cosmópolis	63 639.80	1540.26	Tuiuti	642.38	15.55
Santa Gertrudes	60 232.49	1457.79	Atibaia	321.18	7.77
Santa Maria da Serra	55 252.15	1337.26	Pinhalzinho	321.14	7.77
São Sebastião da Grama	53 582.02	1296.83	Águas de São Pedro	137.11	3.32
Sta Cruz da Conceição	47 763.29	1156.01	Águas de Lindóia	0	0.00
Conchal	45 782.66	1108.07	Bom Jesus dos Perdões	0	0.00
São José do Rio Pardo	44 145.40	1068.44	Campo Limpo Pta	0	0.00
Artur Nogueira	41 827.02	1012.33	Hortolândia	0	0.00
Amparo	41 355.60	1000.92	Lindóia	0	0.00
Tapiratiba	39 447.14	954.73	Louveira	0	0.00
Sumaré	38 354.84	928.29	Várzea Paulista	0	0.00
Total			170 382.54 (TJ year^−1^)		

However, by comparing Tables 1 and 7, it is possible to list the 10 municipalities that have higher positive balances of EE and the ten with negative balances (Table 8).

**Table 8:** The 10 municipalities with the highest positive and negative balances

Ranking	Municipalities	Positive balance (TJ year^−1^)	Municipalities	Negative balance (GJ year^−1^)
1	Brotas	13 431.89	Campinas	−11 175.53
2	Mogi Guaçú	8981.70	Jundiaí	−7116.34
3	Casa Branca	7899.73	Americana	−3720.57
4	Araras	5736.18	Paulínia	−2993.19
5	Pirassununga	5534.86	Sumaré	−2466.12
6	Piracicaba	5126.25	Indaiatuba	−2398.52
7	Capivari	4682.76	Hortolândia	−2004.04
8	Itirapina	4657.55	Bragança Paulista	−1740.61
9	Mococa	4279.27	Valinhos	−1674.88
10	Aguaí	4213.60	Atibaia	−1500.11
	Total	64 543.80	Total	−36 789.92

With the leftover electricity on one side (positive balance of 64 543.80 TJ), it is possible to supply the consumption of the ten major consumers (negative balance of 36 789.92 TJ); that is, approximately 60% more energy is generated. The top 8 consumer municipalities (46 146.85 TJ) have figures (Table 1) that reach almost as high as the consumption of the remaining 82 (47 114.56 TJ). This result shows that both scenarios can be analyzed in terms of public policies. One of the scenarios is aimed at addressing the 8 major consumers, and the other scenario aims at compensating for the remaining 82 municipalities.

### 3.2 Consumption and demand balance: ethanol analysis by municipality

Regarding the residues for bioethanol production, there is a positive balance of 4 × 10^9^ L after discounting the needs of each one of the municipalities. This figure can supply the needs of those that, through their production‐consumption cycle, produced negative figures (1 × 10^9^ L). Thus, the region would be ethanol self‐sufficient when only considering the sugarcane residues and the forestry residues. The region that has intense agriculture also produces residues from annual crops of soybeans, wheat, and beans, which have not been considered in this study.

Table 9 shows the ten municipalities with a positive balance (generation greater than consumption), and they can supply 3 710.54 ML, which is a threefold deficit from that presented by all the municipalities (31). These 31 municipalities have presented a deficit of −965.99 ML, as noted in Table 10. In fact, only one municipality, MG, could supply the deficit from 31 municipalities.

**Table 9:** The 10 municipalities with a surplus production of ethanol

Ranking	Municipalities	Generation – consumption surplus of bioethanol (ML year^−1^)
1	Mogi Guaçú	1005.86
2	Brotas	843.96
3	Casa Branca	514.44
4	Espírito Santo do Pinhal	362.91
5	Itirapina	297.92
6	Aguaí	209.29
7	Itapira	154.27
8	São Sebastião da Grama	123.89
9	Torrinha	104.16
10	Analândia	93.83
Total		3710.54

**Table 10:** The 31 municipalities with a generation deficit for ethanol

	Municipalities	Generation – consumption deficit of ethanol (ML year^−1^)		Municipalities	Generation – consumption deficit of ethanol (ML year^−1^)
1	Campinas	−282.35	17	Socorro	−8.09
2	Jundiaí	−132.16	18	Jaguariúna	−7.45
3	Americana	−74.80	19	Itupeva	−6.61
4	Limeira	−64.24	20	Águas De Lindóia	−5.52
5	Sumaré	−53.54	21	Rio Claro	−5.33
6	Valinhos	−48.18	22	Divinolândia	−3.57
7	Hortolândia	−47.84	23	Lindóia	−3.45
8	Indaiatuba	−47.37	24	Bom Jesus Dos Perdões	−3.43
9	Atibaia	−41.07	25	Cabreúva	−3.17
10	Paulínia	−25.41	26	Pinhalzinho	−3.15
11	Itatiba	−22.65	27	Águas De São Pedro	−3.11
12	Bragança Paulista	−21.75	28	Serra Negra	−2.28
13	Várzea Paulista	−16.35	29	Vargem	−1.12
14	Nova Odessa	−11.35	30	Caconde	−1.04
15	Campo Limpo Paulista	−10.12	31	Pedreira	–0.30
16	Louveira	−9.17			
Total			−965.99 (ML year^‐1^)		

**Figure 10:**
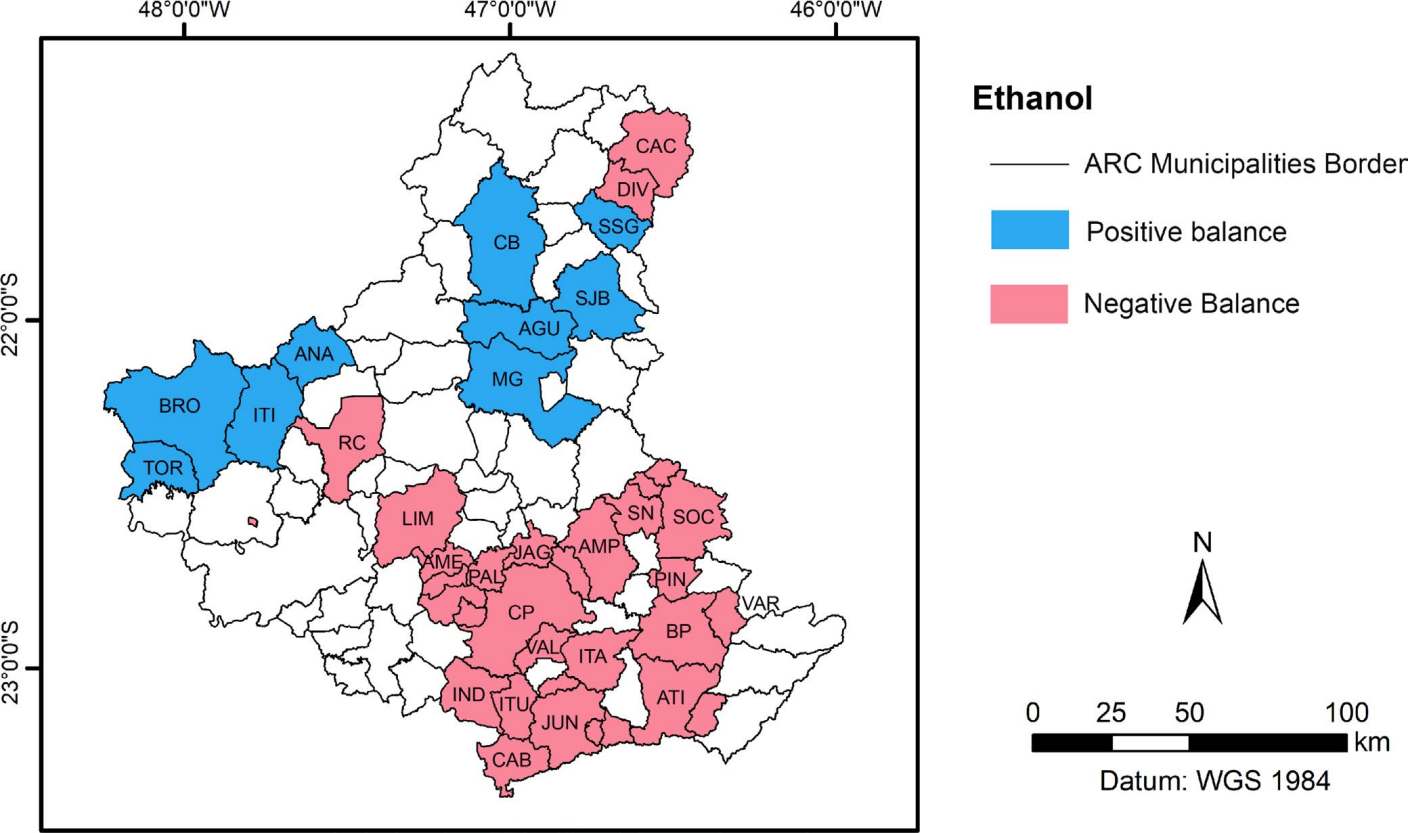
Ethanol map with clear information about the balance between generation and consumption

In terms of public politics, as in the case of ethanol, the map (Figure 10) provides clear information for more direct action, allowing for a better focus on this matter.

## 4 DISCUSSION

Sustainability is important in the context of the bioeconomy or the transition to a bioeconomy, and the time variable and the spatial variable are very important (van Eijck and Romijn, 2008).^65^ Thus, the aim of this study was to contribute tools that may help to reach this goal. The results showed the efficiency of the spatial analysis, and, in this case, the local to regional ranges. Thus, we are on the correct path for residue profits to occur at the local level, and we offer a more appropriate basis for the transition to a bioeconomy as a “local node, global network” Bulkeley.^66^ Furthermore, the results are in accordance with the Brazil iNDC (2014)^6^ primarily concerning GHG mitigation, in implementing policies and measures to adapt to climate change and South‐South initiatives, and in cooperation with other developing countries in areas such as biofuel capacity building, low carbon, and resilient agriculture.

Despite the ARC being located in a region with a well‐built infrastructure, including an energy sector, it has suffered constant variation regarding the hydric conditions that impact electric power generation.^13^, ^15^, ^67^ Some of the needs of the reservoirs that feed the ARC are shared by other important macro regions of São Paulo. This issue causes difficulties in choosing priorities.^68^ The same authors who described the factors that caused the water scarcity in the São Paulo region suggested that the number of days required to produce treated water was increased over the operational limits. Therefore, the amount of water available to customers decreased. Furthermore, this type of situation became more susceptible to extreme climatic events, such as the crises during the summers of 2013/2014 (high temperatures and lack of rain). At this point, new alternatives should be explored to minimize future impacts.^5^

Although our discussion did not focus on social matters, the results may be used to promote greater justice regarding energy access because the spatial analysis describes the local higher or lower residue availability, and as a result, the availability of energy (electric and ethanol) in accordance with some analyses that have had a local/social focus, such as the work of Damgaard et al^69^ We predict that this work will support greater social justice due to the decentralization of biogas generation; however, this goal will require public policies that lead energy companies to take more actions locally. Forbord et al^70^ reinforced the idea that public policies are fundamental to the development of bioenergy at the local and regional levels in cases analyzed in Norway.

The focus of this study was to create conditions for public agents to analyze the energy issue from another perspective in addition to just looking at values. This viewpoint allows for the organization of new policies to consider the residue availability and demand by focusing on local relationships rather than a global perspective. The new trend of thinking about the world, as in Raman and Mohr^71^ and Kline et al,^72^ is that energy and food do not compete; by contrast, they can be complementary in terms of land use, public investments in innovations, technology and rural extension, the promotion of stable prices, and the encouragement of local production.

## 5 CONCLUSIONS

This study focused on estimating the potential for electric energy generation and ethanol production by addressing agricultural residues while considering the spatial analysis. The spatial analysis has shown to be very effective in identifying areas that have agricultural residues, their availability for use as nonfossil fuels and for replacing nonfossil fuels for electrical energy. The balance between the possibility of using those residues to produce electricity and ethanol and their demand in the ARC has allowed us to identify possible ways to exploit that energy, either to feed major consumers (in smaller numbers) or to supply minor consumers (in greater number). Moreover, we explored the synergy by considering the availability of residues (sugarcane and eucalyptus) that could be added to the annual crop residues (not considered in this study) and other important sources of residues to create a more stable set of possibilities for energy generation. This type of initiative will be reflected in hydric (human consumption x agriculture use) and environmental questions (carbon balance and climate change).

In a country with great resources, such as Brazil, this example has demonstrated the benefits of transitioning an economy based on fossil fuels to a bioeconomy. Furthermore, solutions can occur on a local/regional level more than on a national level. Thus, the use of tools for spatial analysis, such as the use of satellite images and geographic information systems, provides great efficiency.
